# Quantitative analysis of degree of substitution/molar substitution of etherified polysaccharide derivatives

**DOI:** 10.1080/15685551.2022.2054118

**Published:** 2022-03-23

**Authors:** Xue-Li Liu, Chun-Feng Zhu, Han-Chun Liu, Jia-Ming Zhu

**Affiliations:** a College of Material and Chemical Engineering, Chuzhou University, Anhui, China; b School of Chemistry & Chemical Engineering, Anhui University, Anhui, China; c Department of Pharmacy, Traditional Chinese Hospital of Lu’an, Anhui, China

**Keywords:** Polysaccharide, etherification, degree of substitution, molar substitution

## Abstract

Due to the unique properties such as nontoxicity, biodegradability, availability from renewable resources, and cost-effectiveness, polysaccharides play a very important part in the science and technology field. The various chemically modified derivatives of these offer a wide range of high value-added in both food and non-food industries. Among the chemical modification, etherified polysaccharide is one of the most widespread derivatives by introducing an ether group which is commonly stable in both acidic and alkaline conditions. Hydroxyalkylation, alkylation, carboxymethylation, cationization, and cyanoethylation are some of the modifications commonly employed to prepare polysaccharides ethers derivatives. There also has been a growing tendency for creating new types of modification by combining the different means of chemical techniques. The correct determination of degree of substitution (DS)/molar substitution (MS) is crucially important. The objective of this article is to summarize developments in synthetic etherified polysaccharides, involving analytical methods for determination of MS/DS, measurement processes, and the associated mechanisms.

## 1. Introduction

In recent years, the requirement for polysaccharide derivatives for various uses has increased dramatically. There are a great many factors contributing to these increases include: (1) There are inexpensive and readily available sources of polysaccharides all over the world, especially for starch, cellulose, and glycogen; (2) polysaccharides have good compatibility with numerous other substances during production, especially hydrocolloid in foods; and (3) polysaccharides are renewable, eco-friendly, and biodegradable. Indeed, on a global scale, intensive efforts are concentrated to produce different kinds of polysaccharide derivatives for different applications that apply to different industries.

Due to the disadvantages of native polysaccharides that limit their use in both food and non-food applications, the modification by chemical or physical techniques is necessary [1]. The various modification technologies, particularly chemical and physical modifications, can alter the properties of different polysaccharide derivatives and their pastes and gels in different ways. Thereinto, the functional properties of native polysaccharides can be improved by chemical modifications, including but not limited to esterification [2], etherification [3–5], acylation [6], crosslinking [7], oxidization [8], and depolymerization by acid [9] or enzymatic hydrolysis [10].

Modifying polysaccharide by etherification significantly changes the physicochemical properties, for example, hydroxypropylation can effectively inhibit the ordered structure of starch paste, retard the retrogradation, enhance the fluidity, and improve the clarity [11]; quaternization acquires higher solubility, better heavy anion-exchange capacity and metal ions sorption [12], alkylation stimulates the benzo[a]pyrene (BaP) aqueous solubilization, and presents high surfactant properties [13]. Furthermore, the specific characteristics of the polysaccharides and the levels of chemical modification are closely related. [14–16] Therefore, it is necessary to determine the extent of etherification as well as the distribution of substituents. Moreover, with the increasing industrial importance of modified polysaccharides, the interest in methods for its analysis is growing.

Normally, there are two parameters which are used to represent the amount of derivatization [14–15]. Simplistically, for now, we will take starch for example. One is degree of substitution (DS), i.e., the average number of hydroxyl groups per repeating unit that were replaced by a given substituent, which is defined by the amount of hydroxyl groups in the repeating unit that can be chemically modified. Hence, the maximum DS varies with the structures of polysaccharides, which is restricted by the total amount of hydroxyl groups that are available within the repeating unit. The maximum DS for starch, cellulose, and glycogen is 3, however, the maximum DS for agarose and xylan is 4 and 2. Some substituents, like hydroxy alkyl groups, feature a hydroxyl group themselves that is also accessible for chemical modification. In these cases, an additional descriptor is required, i.e., molar substitution (MS). The MS value describes the average number of substituents per repeating unit that were introduced. This value can exceed the total amount of hydroxyl groups per repeating unit. The parameter of MS depends on the synthesis conditions of etherified polysaccharide and can vary over a wide range. MS is associated with the degree of side chain formed; the size of the MS value can be theoretically infinite. Among the hydroxyalkylation, hydroxyethyl starch (HES), for example, the hydroxyl group of each hydroxyethyl group can be hydroxyethylated, even multiple etherifications. Thus, the MS is often used to define the formed chains of substituent groups and is used hereafter. So, the MS is the correct term to use for the formed chains of substituent groups and is used hereafter. Meanwhile, the convenient and practical methods are essential to the determination of DS/MS.

Along with the rocketing development of carbohydrate chemistry in the past several decades, a variety of modification strategies and techniques have been discovered and successfully applied and the determination of DS/MS has become a problem that cannot be ignored. A number of review articles concentrated entirely on preparation, characterization of physicochemical properties and application of etherified polysaccharide [16–22], and there is almost no comprehensive review of this topic in recent years. An earlier extensive review by Morgan in 1946 [23] was directed toward the development and improvements of previous methods for the determination of ethylene glycol ethers. In so doing, his research helped pave the way for many valuable explorations in the study of glycol ethers, including hydroxyethyl cellulose. Numerous examples of the use and modified of Zeisel, spectrophotometric, and other methods for the determination of the alkoxyl substituent in polysaccharide were summarized by Cobbler and Samsel in 1962 [24]. Mini-reviews of determination of the hydroxypropyl (HP) level in modified cellulose and starch were surveyed by Ho and Seib, respectively [25,26]. More recently, the methods of determination of amounts of hydroxypropylation were summarized concisely by Fu in 2019 [27], and the determination of the DS of starch esters was outlined by Shi in the ‘DS determination’ section of Part three of the article [1].

Due to insufficient attention paid by the published literature reviews of the comprehensive determination methods of the extent of etherification, we decided to summarize the analytical methods for determination of MS/DS of etherified polysaccharide, including the alkyl ethers, hydroxyalkyl ethers, carboxymethyl ethers, cyanoethyl ethers, cationic ethers, and mixed ethers. Throughout the paper, a systematically discussion of measurement processes and associated mechanisms is also presented. The objective is to enlighten the researchers to identify challenges and opportunities related to this field.

## 2. Hydroxyalkyl ether

**Figure 1.**
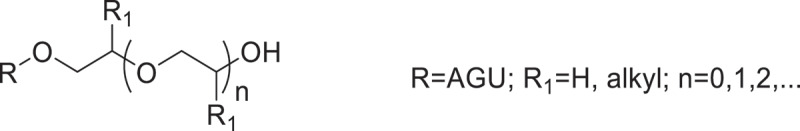
The general formula of some the hydroxyalkyl ethers.

The hydroxyalkylation of polysaccharide involves one or more hydroxyl groups on an anhydroglucose unit (AGU) reacting with epoxides in alkaline conditions. Hydroxyalkyl ethers have (Figure 1) been prepared earlier using ethylene oxide (EO), 1,2-propylene oxide (PO), 1,2-butylene oxide (BO) [28,29], and other long-chain 1,2-epoxyalkanes [30]. Starch hydroxyls attack at the least sterically hindered site on the epoxide via a bimolecular nucleophilic (S_N_2) mechanism. Several analytical methods for the determination of the MS of hydroxyalkyl ethers have been developed over the years and is generally mature enough.

### 2.1. Hydroxyethylation

Hydroxyethylation of polysaccharides was most commonly found in hydroxyethyl starch (HES) and hydroxyethyl cellulose (HEC). For example, HES, as a medically plasma volume expander, the MS and DS are the key determining factors of the duration of pharmacologic action, solubility, and stability in water [31]. As we noted earlier, because of multiple substitutions, there is some difference between DS and MS [32].

**Scheme 1.**
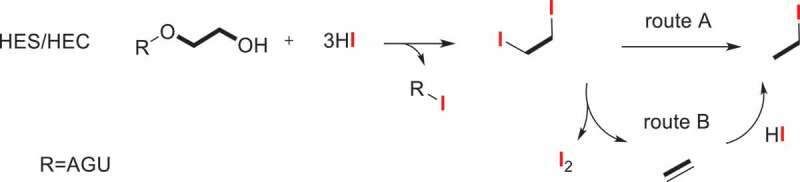
HI-decomposition reaction of HES and HEC.

The previously described method for the analysis of ethoxy group was determined by chemical titration. The measurement of Werner and Mitchell [33] is generally upon the chromic acid oxidation of the ether and titration of the excess dichromate, which was suitable for monomethyl ethers of ethylene glycol. The classical Zeisel method used constant-boiling hydroiodic acid to cleavage the hydroxyethyl ethers into their corresponding alkyl iodides. [34,35] Due to the lack of adequate awareness of another product, i.e., ethylene, it failed to give quantitative and reproducible results in a very long period of time. Until 1946, Morgan [23] devised a modified alkoxyl method and apparatus which was suitable for the determination of hydroxyethyl cellulose via the titration of ethyl iodide and ethylene with silver nitrate and bromine, respectively. In consideration of the feasibility of low substituted hydroxyethyl starch and the amount of excess hydriodic acid, Lortz [36] modified and strengthened the Morgan’s alkoxyl apparatus, adjusted the sample size and corresponding hydriodic acid to regulate the small amounts of ether substitution within the scope of 0.005 to 0.20 hydroxyethyl and hydroxypropyl group per anhydroglucose unit. However, the method is not ideal for alkyl groups of longer chain length than propyl, for instance, hydroxybutyl ether. The absorption-titration method has been used for a long time both here and abroad. In combination with gas chromatographic (GC) technique, several modified Zeisel methods for creating increasingly precise determination for alkyl cellulose ethers, [24,37] hydroxyethyl starch and derivatives [38,39–42] have been published. The analyses of a series of ethyIcelIulose specimens [24], HES 130/0.4 [43,44] and HES 150/0.5 [45] by the gas chromatographic method and the method of chemical titration were compared, the technique of Zeisel gas chromatography provides a simple, rapid and reproducible quantitative analysis method. The proposed mechanism for the hydriodic acid decomposition procedure of HES and HEC is illustrated in Scheme 1. As it is mentioned in the mechanism, there are two routes. One is acid catalyzed and the direct conversion to iodoethane. Another is that the final iodoethane was synthesized via ethylene intermediate. Two routes can lead to the production of iodoethane.

The MS ratio calculation of the HES/HEC is theoretically based on the ethylene oxide unit (C2H4O) by the following equation 1. [18] The parameters *E* and *m* in formula 1 equal the weight of iodoethane and sample; *W_E_* is the weight % of EO in HES; the values 155.97, 162.14, and 44.05 in equation equal the molecular weight of iodoethane, AGU, and C_2_H_4_O.

(1) $$W_{E}=\frac{44.05\times E\times 100}{155.97\times m};MS=\frac{W_{E}}{100-W_{E}}\times \frac{162.14}{44.05}$$

Furthermore, in consideration of the systematic errors of the destructive analysis of HES, such as incomplete cleavage or chemical side reactions during acid hydrolysis, etc., a non-destructive, fast and relatively accurate method to estimate MS with minimal and simple sample preparation is crucially important. As we all know, the NMR spectroscopic method is a powerful tool for analyzing polysaccharide structures. In 2015, Moiseev and co-workers [46] reported the modified Proton Magnetic Resonance (PMR) spectroscopy technique which was used for determining the MS in HES. The results suggested that the PMR spectroscopy is a sensitive and accurate technique with some advantages by correlating the integrated intensities of resonances for terminal anomeric protons, substituted branching chains and unsubstituted AGU residues in HES. In allusion to the identification and quantitative analysis of impurities, it also has certain advantages.

On the other side of the coin, for the most natural polysaccharides mainly of starch and cellulose, the site where the chemical modification occurs, would at its C-2, C-3, and C-6 hydroxyl groups of the AGU to generate the esters, ethers, carbonates or carbamates, etc. The determination of DS and regioselectivity of these which are vitally important for the characterization of original and technologically advanced materials can often be a complex task [47]. For hydroxyethylation, take the HES for example again, the DS of HES has been to shown to be an important measure for determining the efficacy of different starches [32–50]. For the modification of glucose unit, different substitution degree ratios on O-2, O-3, and O-6 have been published [51–54]. The substituent distribution of hydroxyethyl starch has been determined by capillary GC [52] or GLC [55] or GC-MS [41–54] of the silylated hydrolysate with the exception of the early joint use of paper and thin-layer chromatography and spectrophotometry [51].

### 2.2. Hydroxypropylation

**Figure 2.**
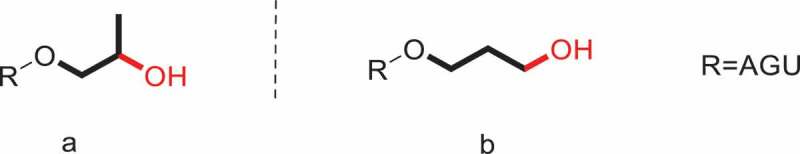
Classical structure (a) and new-type (b)of HPS.

Hydroxypropylation is a widely used and accepted means to modify the structure and properties and promote the functionality of polysaccharides such as chitosan [56–58], starch [59,60], cellulose [61,62], and others [63,64]. It was effective in improving shelf life, freeze-thaw stability, cold water swelling, and reconstituting characteristics. Take Hydroxypropyl starch (HPS), for example. By reacting with PO, the hydroxypropyl groups were introduced into the glucose unit of alkali-activated starch. As a result, the retrogradation has been prevented, the paste clarity has been improved, and the shelf-life, freeze-thaw stability, and cold storage stability to starch-based food products have been extended. The extent of substitution is a key element which influenced the changed physicochemical properties. Several methods used to determine DS/MS have been proposed. In this section, the classical HPS (Figure 2a) and a new-type of HPS (Figure 2b) will be discussed separately (Figure 2).

#### 2.2.1. Classical HPS

There are significant numbers of previous researches on hydroxypropylation of starch with PO. In other words, the hydroxyl group on C-2 of hydroxypropyl rather than the terminal position, namely, β-HPS (Figure 2a). Due to the stability of ether groups in acid and alkali circumstance, β-HPS is a class of modified starch which is widely used in food industry. Meanwhile, the FDA stipulates that all of the hydroxypropylated starches fit the maximum permissible level limitation in food applications, that is to say, the MS should not be more than 0.2 [65]. And for β-HPS, the spectrophotometric (colorimetric) method of Johnson [66,67] is used to determine the MS of hydroxypropyl group and is also an official standard method of the Joint FAO/WHO Expert Committee on Food Additives.

**Scheme 2.**
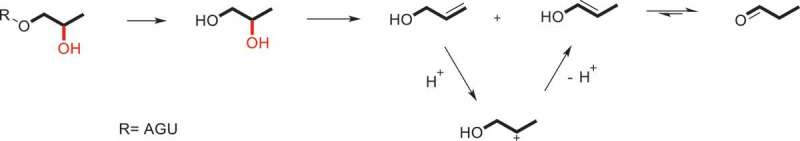
The hydrolysis and dehydration processes of β-HPS.

The colorimetric method is based on reaction of ninhydrin with propanal which is liberated from the HPS during an acid digest, that is to say, involving the dehydration of 1,2-propanediol which is generated from the hydrolysis of the 2-hydroxypropyl group, and then to propionaldehyde and the enolic tautomer (or form) of propanal, which is an isomer of allyl alcohol. As early as 1957, Jones and Riddick [68] had been reported that the 1,2-propanediol was dehydrated to a mixture of allyl alcohol (2-propen-1-0 l) and the enolic form of propionaldehyde by treatment of concentrated sulfuric acid (Scheme 2), and the mixture of allyl alcohol and propionaldehyde can be measured spectrophotometrically at 595 nm by reacting with ninhydrin to generate a violet-colored complex [69].

A standard curve was made using 1,2-propanediol, and native starch was used as a control [66–70]. By using the following formula 2, the MS was calculated through the spectrophotometer by a conversion constant of 0.7763. The parameters P and m in formula 2 equals the weight of the measured propylene glycol and sample, and F is dilution factor; the values 162.14 and 58.08 in equation equal the molecular weight of AGU and C3H6O;

(2) $$W_{P}=F\times \frac{P}{m}\times 0.7763;MS=\frac{W_{P}}{100-W_{P}}\times \frac{162.14}{58.08}$$

**Scheme 3.**
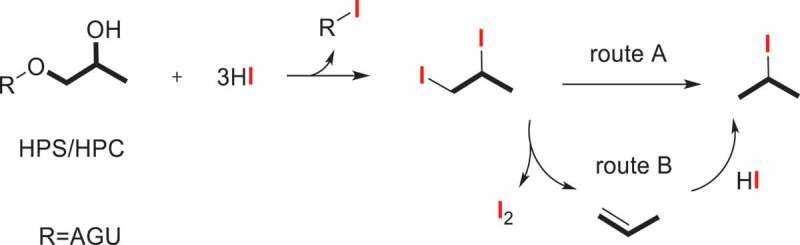
HI-decomposition reaction of HPS/HPC.

Another method is derived from Zeisel determination. In the early stages, the determination of MS of polysaccharide derivatives was mostly using the Morgan method [23] which had been identified as a standard method for testing hydroxyethyl cellulose (HEC) by ASTM [71] in 1976 with some modification by Lortz [36]. The apparatus, operating conditions, and calculation methods had been improved for purpose of making it suitable for the determination of hydroxypropyl ethers. Wang [72] and Xiang [73] modified the classical Morgan method and improved the reproducibility (RSD<1%). As gas chromatography develops, the new improvement in separation and determination of the alkyl halides become possible. In 1962, Cobler and Samsel [24] investigated the 3-isopropoxy-n-propylamine and hydroxypropyl cellulose (HPC) ethers by the modified Zeisel method with gas chromatographic analysis which affords a 50% time saving. In 1979, an improved Zeisel gas chromatographic technique had been reported for the determination of MS in HPC by Hodges [37]. The experimental and calibration of HPC were determined and a proposed mechanism for the HI-decomposition procedure of HPC was illustrated in Scheme 3.

**Figure 3.**
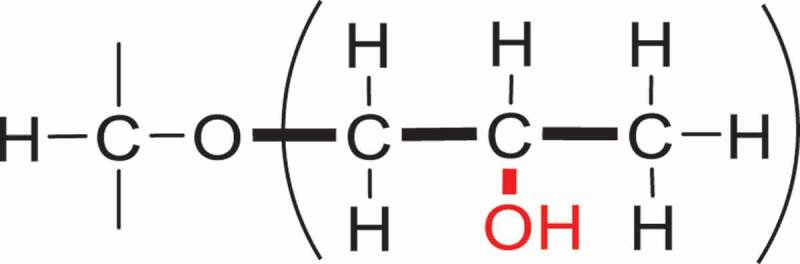
Classical structure of the hydroxypropyl group.

FTIR spectroscopy was used to detect hydroxypropylation in modified starches. It can not only characterize the structure of the HPS, but also estimate the degree of substitution by using the derivative difference spectroscopy. In the past, IR spectroscopy had been used to estimate the degree of substitution of various modified starches (e.g., cyanoethyl starch, acetate starch, sulphate starch) [74] and pectin [75]. Due to limitations of the instrumentation, FTIR spectroscopy was used to detect hydroxypropylation in HPS successfully until 1992 [76]. The hydroxypropyl substitution can be detected in a modified starch by IR. As is shown in Figure 3, compared with the other functional groups, the methyl group is the only distinctive characteristic which observed as a peak of asymmetric methyl C-H stretching centred at 2974 cm^−1^. The magnitude of the peak varies according to the contents of HP substitution in the starch, and the peak is clearly defined. The method provides a rapid means of detecting hydroxypropylation by using the second derivative difference spectrum of the HP group. The area under the peak centered at 2974 cm^−1^ is calculated, and the content of HP group is measured by utilizing a calibration curve. The studies demonstrate a correlation between the content of HP substitution and the spectral property in the mid infra-red region. It would be a means of rapid quantitation once there were characteristic peaks and a reliable set of secondary standards.

**Figure 4.**
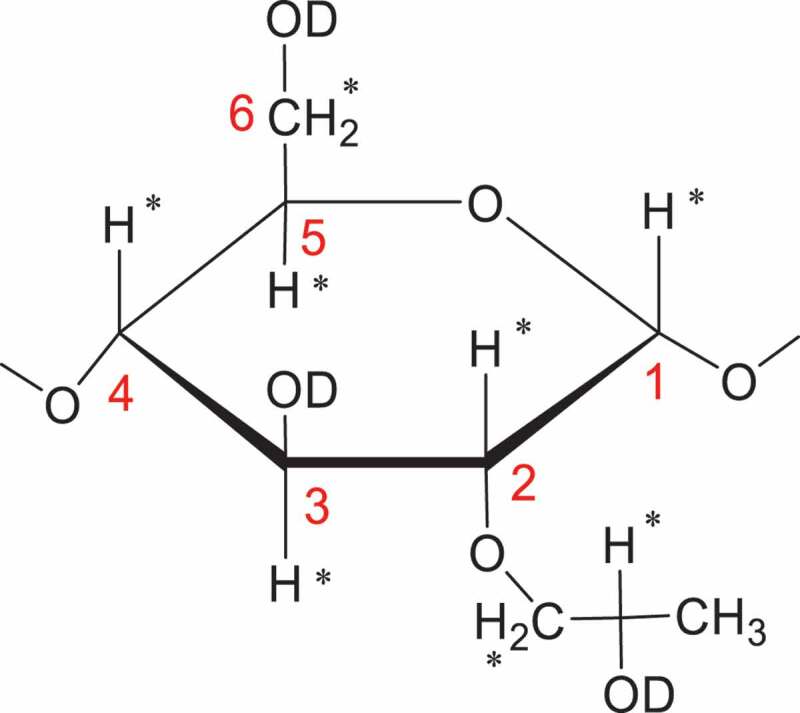
The structure of a deuterated AGU substituted with one HP group on O-2.

Last but not least to mention is that the proton nuclear magnetic resonance [1H-NMR] spectroscopy can effectively measure the relative contents of HP groups and glucose units [77–79]. As with FTIR spectroscopy, it is also utilizing the ratio of the respective proton integrals between the chemical shifts of the methyl group protons of the HP group and the protons of the polysaccharides. The [1]H-NMR method is a two-step method, comprising of depolymerization and deuteration. The purpose of the former process is to cut down the molecular weight of the polysaccharides with the treatment of acid-catalyzed hydrolysis [77–80] or α-amylase-catalyzed hydrolysis [26–83]. After the former hydrolysis process, all hydroxyl group protons were exchanged with deuterium by treatment with a right amount of D2O added into the NMR tube (Figure 4).

There are two general approaches in which the HP contents of HPS could be calculated. One approach uses the known concentrations of CH_3_COOH (or CH_3_COONa) as an external standard, so, the integrated intensities of protons on the HP methyl group (I_HP_) and acetyl (I_Ac_) could be utilized to determine the MS directly with the following equation 3, where *m* and *I,* respectively, represent the weight and comprehensive peak resonance integrated areas of the resonances assigned to the methyl groups of HP and acetic acid.

(3) $$W_{P}=\frac{58.08\times I_{HP}\times m_{NaOAc.3H_{2}O}\times 100}{136.08\times I_{Ac}\times m_{Starch}};MS=\frac{W_{P}}{100-W_{P}}\times \frac{162.14}{58.08}\;$$

The other approach utilizes the comprehensive area of the six protons on C-2, C-3, C-4, C-5, and C6 of a glucosyl unit other than hydroxyl group protons [80,81] or the anomeric proton [78] to calculate MS. With the former, the MS can be figured up by using the proportion of the integrated intensities of protons on HP substituents and oxygenated carbons (HCO) of the AGU, including the methylene (C-6) and methine (C-2, C-3, C-4, C-5) protons to the integrated intensity of the HP methyl signals without regard to the anomeric proton (Equation 4). For the latter, the anomeric proton of the AGU of starch was used as an internal standard; the MS can be counted up directly from equation 5. The symbol *I_HP_* in Equations (4) and (5) is the integrated intensity of the methyl group (-CH3) on HP substituents, and the symbol *I_HAP_* is the integrated intensity of the anomeric (C-1) proton of the AGU, and *I_HCO_* is the integrated intensity of all the methylene (C-6) and methine (C-2, C-3, C-4, C-5) protons on the AGUs.

(4) $$MS=\frac{2\times I_{HP}}{I_{HCO}-I_{HP}}$$

(5) $$MS=\frac{I_{HP}}{3\times I_{HAP}}$$

As for hydroxyalkyl etherified starch, the incidence of reaction is known to occur mainly at the hydroxy group on O-2 by reacting with EO and PO in base [26–84]. The anomeric region of the AGU in starch and starch hydrolysis products have been distinguished previously by Gidley through the use of the [1] H-NMR spectra [85]. As for the distribution of HP groups on O-2, O-3, and O-6 of a glucosyl unit, Xu and Seib [26] determined the distribution of HP groups of several starch ethers with DS values ranging from 0.05 to 0.23 by [1] H-NMR, and concluded that the probability of HP substitution was 67%–78% on O-2, 15%–29% on O-3 and 2%–17% on O-6. With increasing the DS, the proportion (%) of O-6 leaned to rise slightly; for the O-3, however, tended to decrease slightly. Only the O-2 remained essentially unchanged. All of the conclusions are consistent with what has been found with cellulose and other starch ethers [52–88].

#### 2.2.2. New-type HPS

The HPS described above is β-HPS, i.e., the hydroxyl group on C2 of hydroxypropyl rather than the terminal carbon atom (Figure 2a). A new type of HPS, γ-hydroxypropyl starch (γ-HPS), is prepared by employing 3-chloropropanol as the etherifying reagent [89]. The analogous-structured γ-HPS involving the hydroxyl group on the terminal C3 position of propyl group (Figure 2b) was reported by our group. Two independent measurements for the determination of MS in γ-HPS were described.

**Scheme 4.**
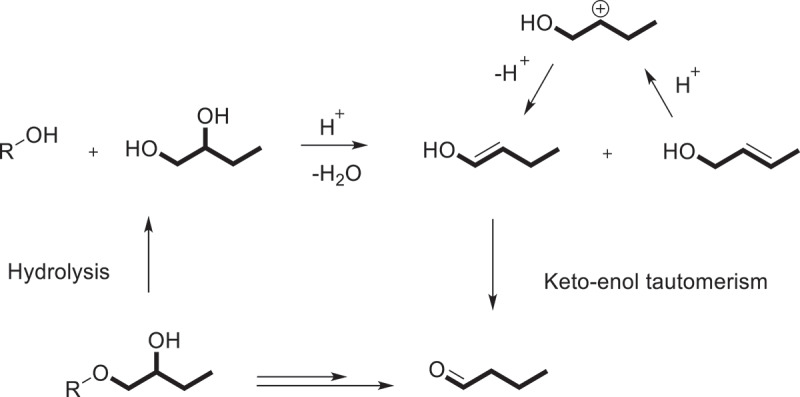
Proposed mechanism of the butyraldehyde generation from HBS.

One is colorimetric Method. In order to explore the feasibility, the classical spectrophotometric method of Johnson was measured. A calibration curve with 1,3-propanediol was prepared, and the wavelength optimum, reaction time and precision determination were investigated. Due to the result and the literature [68], an improved colorimetric process was presented with a good repeatability (RSD = 0.37%) which was appropriate for determination of γ-HPS. The proposed mechanisms of two colorimetric processes were illustrated in Scheme 4. The MS ratio was calculated by the equation (2).

Another is Zeisel-Gas Chromatography with a feasible mechanism for the degradation reaction through an assumed 1,3-diiodo intermediate. The process of the cleavage reaction could be partially understood by chromatographing the reaction products during the course of the experiment. The peak area ratio of 2-iodopropane (A)/ 1-iodopropane (B) is over 40:1, which illustrated that 2-iodopropane is the major hydrolysis product. The actual quantity of final hydrolysis product can be figured up by gathering A and B. Calculation of the MS of γ-HPS is theoretically based on the propyl oxide unit, C3H6O, by the following equation 6. Refer to the HES, the P and m in formula 5 equal the weight of iodopropane and the sample of γ-HPS; The value of number 58.08, 169.99, and 162.14 were represented the molar mass of C_3_H_6_O, iodopropane, and AGU.

(6) $$W_{P}=\frac{58.08\times P\times 100}{169.99\times m};MS=\frac{W_{P}}{100-W_{P}}\times \frac{162.14}{58.08}$$

### 2.3. Hydroxybutylation

**Figure 5.**
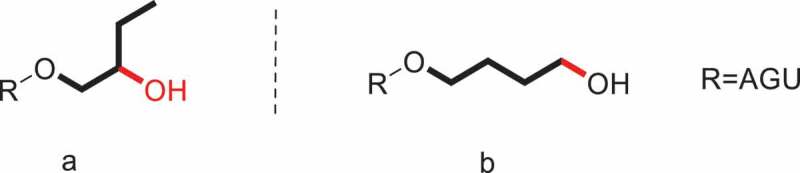
Classical structures (a) and new-type (b) of HBS.

Hydroxybutylation is also an important way to attain polysaccharides modification. Currently published literatures on hydroxybutyl-modified polysaccharides are more common in chemical modification of chitosan [90–93]. For starch, the HES and HPS possess many advantages, thus great attention has been paid to their development and application by scholars both at home and abroad in recent years; but there is scarce study on the hydroxybutyl starch (HBS) which is part of nonionic starch ether [94–96]. Good hydrophilicity and stability and favorable thermo-responsive property could be obtained by hydroxybutylation. Just like HPS, the classical HBS and a new-type of HBS will be stated and discussed separately in this section (Figure 5).

#### 2.3.1. Classical HBS

The classical HBS was synthesized by utilizing 1,2-epoxybutane as hydroxylalkylation reagent in aqueous NaOH (Figure 5a). The most frequently used method for the determination of the MS of HBS was ultraviolet spectrophotometry which was published by Harry-O’Kuru [97]. This whole conversion process contains two parts illustrated below in Scheme 4. The first stage in conversion is hydrolysis involving the generation of 1,2-butanediol; the intermediate 1,2-butanediol will be turned into n-butyraldehyde and the enolic form of butenol by dehydration in the second step (Scheme 4). Meanwhile, both of the absorbance maximum and standard curves between absorbances and concentrations proved the feasibility of the method for the determination of the MS of HBS. All of the findings of the research provided basis for spectrophotometric procedure for estimating the MS of HBS. The method is simple, low cost, high accuracy, and can be used as a conventional method in industrial application.

#### 2.3.2. New-type HBS

In recent years, there have been some researches about the synthesis and characterization of 2-hydroxybutyl starch, however, the new style of δ-hydroxybutyl starch (δ-HBS), i.e., the hydroxyl group in the terminal of butyl was rarely reported (Figure 5b). Owning to the difference in chemical structure and method feasibility [68], the classical spectrophotometric method was not applicable to δ-HBS. Herein, an improved Zeisel gas chromatography for the estimation of the MS of δ-HBS was described [98].

Different from the cleavage of HES/HEC or HPS/HPC [38–42], the procedure of displacement reaction of δ-HBS was a little bit complicated. 1-Iodobutane and 2-iodobutane are the end products of HI-decomposition procedure. A few control reactions were conducted, and the latter is the major degradation product. As we stated before, the generated diiodide intermediate initially could be converted to the final iodobutane through two kinds of routes. Either of these routes can lead to the production of 2-iodobutane.

## 3. Alkyl ether

Etherification of native polysaccharides is generally obtained by reacting hydroxy or amino groups with alkyl halides, acrylonitrile, or epoxy alkanes in the presence of an alkaline catalyst. The various functional groups introduced with AGU substitution may be either hydrophobic or hydrophilic and they increase or decrease hydrophilicity of the modified polysaccharides. One of the typical hydrophobic functional groups is the alkyl or benzyl group, which is present in organic ethers of polysaccharides (cellulose [99], starch [100,101], chitin [102,103], and pullulan [104] under basic conditions. Increasing the substitution level with alkyl group markedly increases hydrophobicity of alkyl polysaccharide ethers.

According to different etherification substrates, several methods used to determine DS/MS have been proposed. For alkylation of chitosan, the DS per glucosamine unit was often determined by titration [105,106], C/N ratio of elemental analysis and NMR analysis [103].

With the first approach, the extent of reaction and DS determination are derived by analyzing the change of bromide ions, that is to say, the generation rate of bromide ions is the conversion rate of etherification, and the DS of ethers can be calculated by the equation 7 further [106], where *M* is molar mass of the alkyl group; *c* is the concentration of silver nitrate standard solution (mol/L); *V* is the volume of consuming silver nitrate standard solution (mL); m is the weight of ethers.

(7) $$DS=\frac{162.14\times c\times V}{m-M\times c\times V}$$

The second method is specific to particular chitosan and its derivatives. The DS was calculated from the C/N ratio of elemental analysis, on account of a particular element nitrogen [102–[107–110]. And finally, the method of NMR analysis is suitable for nearly all the polysaccharides and derivatives with introduction of groups which have specific characteristic peak. For instance, the methyl and methylene of the propyl group from propyl-etherified amylose [101], the methylene of the allyl group from allyl chitosan derivatives [103], the methylene of the cyanoethyl group from cyanoethyl chitosan [111]. DS was calculated from the peak area of the NMR spectrum by measuring and comparing the integral areas of proton signals in structural fragments. Reference is also made to the ‘2.2.1’ section in this article.

## 4. Other ether

Polysaccharides are typically the natural products. After a series of complicated chemical reactions, involving carbon-capture process, photosynthesis and more complex biosynthetic modifications, carbohydrates are formed. Therefore, different forms of modifications are applied to optimize the structural and functional properties of polysaccharides to achieve the targeted applications. So, different methods used to modify starch characteristics include enzymatic, physical, or chemical modification. Etherification is one of the most important means for the modifications of polysaccharides. Besides the above-mentioned ethers, we also emphasize other polysaccharide ethers in this section.

### 4.1. Carboxymethyl ether

Modification is commonly done to improve the structural composition, molecular weight, linkage pattern and ionic character of polysaccharides. Carboxymethylation, which can endow macromolecule with outstanding physical and chemical properties, is an important way to introduce carboxyl groups into biopolymers. For example, carboxycelluloses are important derivatives of natural cellulose polymers, and they have been widely used in many biomedical, agricultural, and wastewater treatment field applications [112,113]. It is well known that the properties (viscosity of solution, film forming, interaction with cations, and the formation of supramolecular aggregates, etc.) are mainly determined by the total DS, i.e., the average number of carboxymethyl groups in the polymer [107].

There are many kinds of methods to measure the DS of carboxymethyl polymers in direct or indirect way, including the ashing method [108,109], acid washing method [110–117], spectrophotometry [118], complexometry [119], NMR method [120,121], Zeisel-LC [122], etc [123–127]. In industry the DS is usually determined by titrimetric methods [128]. All of first four methods belong to this. Over the years, aiming at the problem of time-consuming, poor stability and testing effectiveness in classical complexometry, many other improvements have been made by Huang [129], including the replacement for murexide indicator with PAN indicator (1-(2-pyridinylazo)-2-naphthalenol) and adjusting the original pH (7.5–8.0) to slightly acidic (6.0–7.0) for inhibition of precipitation of Cu(OH)2. Like acid–base back titration, this is also one of most popular ways of determining the DS of carboxymethyl ethers. Moreover, the substitution degrees of carboxymethyl groups were further calculated and obtained from their respective [1]H NMR spectra. Due to the protons of the methylene on the carboxymethyl group, a new proton signal appeared in the NMR spectra of all carboxymethyl ethers. Besides, the peak area of the aforementioned new appeared NMR signal turns larger and larger with increasing amount of monochloroacetic acid fed in the preparation process. The contents of carboxymethyl group were calculated on the basis of the integrated areas of the corresponding characteristic peaks in the [1]H NMR spectra (equation 8). Where *A* is peak areas; *N is the* number of protons; *Signal* is assigned to the methene (-CH_2_COOH); *AGU* is the proton of single hydrogen of an anhydroglucose unit. In addition, the extent of functionalization (degree of substitution) in chitosan derivatives was quantitatively assessed using the elemental analyses of burnable. The degrees of substitution (DS) of carboxymethyl chitosan derivatives were calculated on the basis of the percentages of carbon and nitrogen [130].

(8) $$DS=\frac{A_{Signal}/N_{Signal}}{A_{AGU}/N_{AGU}}$$

### 4.2. Cyanoethyl ether

Cyanoethylation of biopolymers such as chitosan [111–132], guaran [133], starch [134–136], bagasse [137], cotton [138], cellulose [139,140] and others [141–143] can be performed by reacting starch with acrylonitrile using Michael addition. The introduction of cyano group into the polysaccharides has for long been used to improve properties of polymers. Take starch, for example, this treatment gives the starch a resistance to biodegradation, a good water solubility, a thick paste in water and adhesive properties [144]. Similarly, the DS has an effect on physicochemical properties of cyanoethyl polysaccharides [111–143]. The extent of cyanoethylation was determined by using the Kjeldahl method of nitrogen determination [136]. The degree of substitution (DS) was calculated by C/N ratio of elemental analysis [145–147]. Due to the methylene linked to nitrile group from cyanoethyl polysaccharides, [1]H-NMR could be employed for further confirmation of the DS [111–148].

### 4.3. Cationic ether

Cationic polysaccharides are generally synthesized by the reaction of polysaccharides and cationic reaction reagents such as tertiary amine compounds, quaternary amine compounds, and imine compounds. Take the starch for instance. Tertiary amine ether starch and quaternary ammonium ether starch are the main commodities starch. They are non-toxic and easily biodegradable. Introducing a cationic group to the starch gives good mineral binding properties; they are widely used in the paper industry, where they are mainly used as a flocculation, dispersion and ink fixing agent [149]. Thus, the DS is an important parameter [150]. The degree of substitution (DS) was calculated from the nitrogen content which was estimated by the micro Kjeldahl method after purification (equation 9) [116–129,132–151]. Where *N%* is the content of nitrogen measured by Kjeldahl; the values 14, 162.14 and *M* in equation equal the molecular weight of Nitrogen element, AGU and *cationic ether*. There are significant parallels with chitosan, cyanoethyl ether, and other nitrogenous compounds.

(9) $$DS=\frac{162.14\times N\%}{14-M\times N\%}$$

## 5. Mixed ethers

Over recent years, for the purpose of better performance and more widespread application, considerable attention has been focused upon the treatment and disposal of the modification of polysaccharides. However, it is difficult to meet the market demand by using a single modification process. There has been a trend to combine different kinds of chemical treatments to create new kinds of modifications. For the moment, more and more often, the polysaccharides modification processes are complex, i.e., combine two reagents or two methods. The physicochemical properties of chemically modified polysaccharides depend on various factors, primarily on the type of modifying agent, the conditions of reaction, and the kind of polysaccharides, as well as the value of DS/MS. To evaluate on the DS/MS, of course, calculate separately on each of the substituent groups. For example, acetylated oxidised starch is one obtained by dual chemical modification [152]. Assessment of the effectiveness of oxidation and acetylation was based on the increase in the contents of carboxyl groups and carbonyl groups [153] and acetyl groups [154] in starch. Beyond that, there are several composite modified polysaccharides, such as acetylation in hydroxypropyl chitosan [155], hydroxypropyl methylcellulose [156–160].

## 6. Conclusions

Polysaccharides are generally undergoing the modification with the aim of satisfying their physicochemical properties to the requirements of the technological processes in which they will be utilized as additives only to ensure the appropriate structural performance and storage stability of the final product. The increasing industrial importance of polysaccharides ethers has aroused interest in methods for their analysis. The value of DS/MS markedly affects the properties of these compounds and the suitable methods for their determination are of great necessity. In conjunction with the research mentioned previously, the aim of this article is to summarize the chemical modification of polysaccharides with etherification, and the measurement processes of DS/MS and associated mechanisms are involved. We look forward to seeing that this review could give a summarization and prospect on analytical method of DS/MS for etherified polysaccharide derivatives and wishing to help encourage further research on new methods of analysis and modification.
